# A Retrospective Study Investigating the Safety and Efficacy of Nanoliposomal Irinotecan in Elderly Patients with Unresectable Pancreatic Cancer

**DOI:** 10.3390/jcm12103477

**Published:** 2023-05-15

**Authors:** Tatsuki Ikoma, Toshihiko Matsumoto, Shogen Boku, Tomoyo Yasuda, Masataka Masuda, Takashi Ito, Koh Nakamaru, So Yamaki, Shinji Nakayama, Daisuke Hashimoto, Tomohisa Yamamoto, Nobuhiro Shibata, Tsukasa Ikeura, Makoto Naganuma, Sohei Satoi, Takayasu Kurata

**Affiliations:** 1 Cancer Treatment Center, Kansai Medical University Hospital, 2-3-1, Shinmachi, Hirakata 573-1191, Osaka, Japan; 2 Department of Thoracic Oncology, Kansai Medical University, 2-3-1, Shinmachi, Hirakata 573-1191, Osaka, Japan; 3 Department of Gastroenterology, Kansai Medical University, 2-3-1, Shinmachi, Hirakata 573-1191, Osaka, Japan; 4 Department of Surgery, Kansai Medical University, 2-3-1, Shinmachi, Hirakata 573-1191, Osaka, Japan; 5 Division of Surgical Oncology, University of Colorado Anschutz Medical Campus, Aurora, CO 80045, USA

**Keywords:** nanoliposomal irinotecan (nal-IRI), pancreatic cancer, elderly patients, nutritional status

## Abstract

Although nanoliposomal irinotecan combined with 5-fluorouracil and leucovorin (nal-IRI+5-FU/LV) has been used to treat first-line resistant unresectable pancreatic cancer, the efficacy and safety data among the elderly remain limited. We retrospectively analyzed clinical outcomes among elderly patients. Patients treated with nal-IRI+5-FU/LV were assigned to the elderly (≥75 years) and non-elderly (<75 years) groups. Herein, 85 patients received nal-IRI+5-FU/LV, with 32 assigned to the elderly group. Patient characteristics in the elderly and non-elderly groups were as follows: age: 78.5 (75–88)/71 (48–74), male: 17/32 (53%/60%), performance status (ECOG) 0:9/20 (28%/38%), nal-IRI+5-FU/LV in second line: 23/24 (72%/45%), respectively. A significantly high number of elderly patients exhibited aggravated kidney and hepatic functions. Median overall survival (OS) and progression-free survival (PFS) in the elderly group vs. non-elderly group were 9.4 months vs. 9.9 months (hazard ratio (HR) 1.51, 95% confidence interval (CI) 0.85–2.67, *p* = 0.16) and 3.4 months vs. 3.7 months (HR 1.41, 95% CI 0.86–2.32, *p* = 0.17). Both groups exhibited a similar incidence of efficacy and adverse events. There were no significant differences in OS and PFS between groups. We analyzed the C-reactive protein/albumin ratio (CAR) and neutrophil/lymphocyte ratio (NLR) as indicators that could determine eligibility for nal-IRI+5-FU/LV. The median CAR and NLR scores in the ineligible group were 1.17 and 4.23 (*p* < 0.001 and *p* = 0.018, respectively). Elderly patients with worse CAR and NLR score could be deemed ineligible for nal-IRI+5-FU/LV.

## 1. Introduction

Pancreatic cancer (PC) is well-associated with one of the worst prognoses and is often detected in the metastatic state [1]. In addition, it has been reported that the median age at diagnosis is ≥70 years [2]. Accordingly, PC among elderly patients remains an urgent challenge. Chemotherapy is considered the standard therapy for unresectable PC (URPC), regardless of age. Regimens such as FOLFIRINOX or gemcitabine-based therapy are employed as first-line therapy for URPC, and most elderly patients undergo gemcitabine-based therapy, considering tolerability [3,4]. After first-line therapy, nanoliposomal irinotecan (nal-IRI) combined with 5-fluorouracil (5-FU) and leucovorin (LV) has been widely employed, based on the results of the NAPOLI-1 trial [5]. 

The NAPOLI-1 trial was a global phase III trial assessing patients with URPC after gemcitabine-based therapy, comparing nal-IRI combined with 5-FU and LV (nal-IRI+5-FU/LV) and 5-FU/LV groups. The median overall survival (OS), the primary endpoint, was significantly improved in the nal-IRI+5-FU/LV group when compared with that of the 5-FU/LV group, thereby confirming its efficacy in URPC after previous gemcitabine-based therapy. Elderly patients >75 years of age were also included in the NAPOLI-1 trial, with a report in the elderly subgroup [2]. The authors found that both elderly and young patients benefited from nal-IRI+5-FU/LV therapy. However, in the NAPOLI-1 trial, only 14 patients aged >75 years underwent nal-IRI +5-FU/LV therapy [2]. The trial did not include sufficient cases to confirm the safety and efficacy among elderly patients >75 years. 

Therefore, in the present retrospective study, we examined the efficacy and safety of nal-IRI+5-FU/LV in elderly patients aged >75 years in a real-world clinical setting. Moreover, switching to best supportive care (BSC) rather than chemotherapy may be more suitable in specific elderly patients, considering the general condition of patients. However, it has been reported that BSC is frequently selected based on age alone in clinical settings, and patients who were eligible for chemotherapy were frequently switched to BSC [6]. Establishing the eligibility of elderly patients for chemotherapy remains a critical challenge. Therefore, we also examined potential indicators to detect patients who would be ineligible for nal-IRI+5-FU/LV and analyzed the value of inflammatory nutritional factors in our study to determine relevant patients [7].

## 2. Materials and Methods

### 2.1. Study Design and Patients’ Characteristics

Clinical data of consecutive patients with URPC treated with nal-IRI+5-FU/LV as second-line or later treatment were retrospectively collected from Kansai Medical University Hospital. Eligible patients were ≥18 years; had locally advanced, recurrent, or metastatic PC; and underwent at least one cycle of nal-IRI+5-FU/LV between March 2020 and July 2022. Patient data were evaluated from the date of registration until September 2022. The patients were administered nal-IRI 70 mg/m^2^, 5-FU 2400 mg/m^2^, and LV 400 mg/m^2^ every two weeks until tumor progression or intolerance developed. Doses were reduced according to a clinical trial [5]. We defined “elderly” patients as those aged ≥75 years. The present study was conducted in accordance with the Helsinki Declaration of 1964 and its later versions and with the ethical guidelines for clinical studies. This study was approved by the institutional review board of Kansai Medical University (approval no. 2022219). 

### 2.2. Definition of Inflammatory Nutritional Factors and Organ Function

We examined three inflammatory factors, including neutrophil/lymphocyte ratio (NLR), platelet/lymphocyte ratio (PLR), and C-reactive protein (CRP)/albumin (Alb) ratio (CAR) [8,9,10]. These factors were determined based on blood tests performed on the date of first initiating nal-IRI+5-FU/LV. NLR was defined as the absolute neutrophil count divided by the absolute lymphocyte count. PLR was defined as the absolute platelet count divided by the absolute lymphocyte count. CAR was measured by dividing the serum CRP value by the serum Alb value. We analyzed two indicators for assessing organ function: creatinine clearance (CCR) and the bilirubin score (ALBI score). CCR and ALBI scores are indicators of renal and hepatic function [11,12]. CCR is widely used as an indicator for assessing renal function in daily practice, as well as for establishing chemotherapy doses. In recent years, the ALBI score has also been widely used as an indicator of hepatic reserve capacity in hepatocellular carcinoma [13]; hence, this score was adopted in the present study [14]. In addition, patients with a survival of <3 months were defined as the population in which the nal-IRI+5-FU/LV had no prognostic value, indicating that an OS < 3 months resulted in ineligibility for chemotherapy.

### 2.3. Statistical Analysis

OS was defined as the time from the date of first initiating nal-IRI+5-FU/LV to the date of death. The living patients were censored at the last follow-up visit. Progression-free survival (PFS) was defined as the duration from the date of first initiating nal-IRI+5-FU/LV to the date of exacerbation confirmation via computed tomography (CT) or death for any reason. CT-based disease assessment was performed every eight weeks, based on the Response Evaluation Criteria in Solid Tumors version 1.1. Patient characteristics were compared using Fisher’s exact test and Mann–Whitney U test. OS and PFS were estimated using the Kaplan–Meier method. The log-rank test was used to compare groups, whereas the Cox regression model was used to calculate hazard ratios (HR) and 95% confidence intervals (95% CI). A receiver operating characteristic (ROC) curve was used to determine the correlation between OS < 3 months and patient characteristics or nutritional factors, as well as cut-off values for OS < 3 months. The predictive performance was evaluated using the area under the ROC curve (AUC). Patients are frequently excluded from clinical trial participation if the expected prognosis was <3 months. Therefore, in the present study, we followed the same practice and analyzed factors that determined OS for <3 months. Statistical analyses were performed using SPSS software, version 28.0 (IBM Corp., Armonk, NY, USA), and a *p*-value < 0.05 was deemed statistically significant. 

## 3. Results

### 3.1. Patient Characteristics

Between March 2020 and July 2022, 85 consecutive patients with URPC were treated with nal-IRI+5-FU/LV as a second-line or later treatment. These 85 patients were divided into the elderly and non-elderly groups. Elderly patients were defined as those aged ≥ 75 years, i.e., the elderly group (*n* = 32); the non-elderly group (*n* = 53) comprised those <75 years of age. Table 1 summarizes the characteristics of patients in each group. The elderly group had significantly fewer cases of liver metastasis and previous irinotecan use than the non-elderly group (*p* = 0.01 and <0.01). There was no difference in the ECOG PS performance status or other metastatic lesions between groups. A higher number of patients in the elderly group experienced impaired renal and hepatic function than those in the non-elderly group.

### 3.2. Comparison of OS and PFS between the Elderly and Non-Elderly Groups

**Figure 1:**
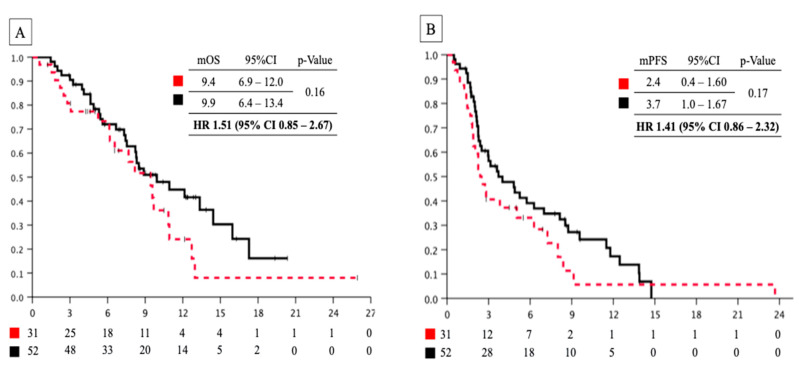
(**A**) Overall survival curve of patients with pancreatic cancer treated with nal-IRI+5-FU/LV between the elderly and non-elderly groups. Red dashed line: elderly group; black line: non-elderly group. (**B**) Progression-free survival curve patients with pancreatic cancer treated with nal-IRI+5-FU/LV between elderly and non-elderly groups. Red dashed line: elderly group; black line: non-elderly group. nal-IRI+5-FU/LV, nanoliposomal irinotecan combined with 5-fluorouracil and leucovorin; mOS, median overall survival; mPFS, median progression-free survival; HR, hazard ratio.

Considering the median observation period of 7.4 (range, 1.2–25.9) months for censored cases, there was no significant difference in median OS between the elderly and non-elderly groups (9.4 (95%CI 6.9–12.0) months vs. 9.9 (95%CI 6.4–13.4) months, HR; 1.51 (95%CI 0.85–2.67); *p* = 0.16) (Figure 1A). Additionally, we noted no significant difference in median PFS between the groups (2.4 (95%CI 0.4–1.60) months vs. 3.7 (95%CI 1.0–1.67) months, HR: 1.41 (95%CI 0.86–2.32); *p* = 0.17) (Figure 1B). We compared the OS between the two groups based on the number of previous treatments and previous irinotecan use. On analyzing patients who had received at least two previous therapies or had previously used irinotecan, we detected no difference between the two groups in the whole population; however, the median OS was significantly worse in elderly patients who had received one previous therapy or had not previously received irinotecan than the non-elderly group (7.7 (95%CI 4.7–10.6) months vs. 14.4 (95%CI 6.4–22.4) months; *p* = 0.003, and 9.4 (95%CI 6.9–11.9) months vs. 12.1 (95%CI 7.3–17.0) months; *p* = 0.043). 

### 3.3. Safety and Efficacy of Nal-IRI+5-FU/LV Therapy in Elderly and Non-Elderly Groups

Table 2 presents the adverse events in the two groups. No adverse events resulted in patient deaths. Moreover, the frequencies of grade 4 hematologic toxicity and grade ≥3 non-hematologic toxicity were similar in elderly and non-elderly groups (19% vs. 8%, *p* = 0.17 and 13% vs. 11%, *p* = 1.00, respectively), with no differences in efficacy observed between groups. The overall response and disease control rates in the elderly and non-elderly groups were 9% vs. 12% and 47% vs. 56%, respectively (*p* = 0.50 and *p* = 1.00, respectively). The elderly group experienced a greater number of dose reductions at initiation than the non-elderly group (47% vs. 11%, *p* < 0.01). 

### 3.4. ROC Curves Analysis for Identifying Eligible Cases in the Elderly Group

**Figure 2:**
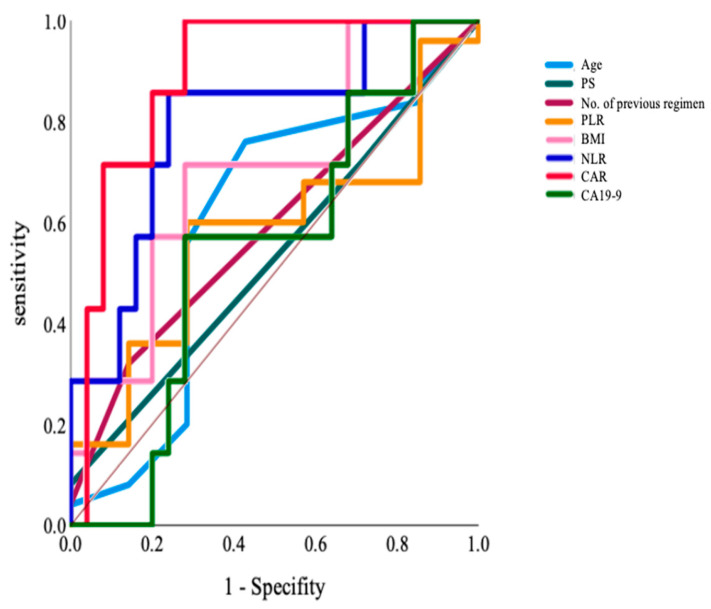
The ROC curve analyzing the superiority index to determine the ineligibility for chemotherapy in the elderly group: Age, ECOG PS, The number of previous regimens, BMI, NLR, PLR, CAR, and CA19-9. BMI, body mass index; CAR, C-reactive protein/albumin ratio; NLR, neutrophil/lymphocyte ratio; PLR, platelet/lymphocyte ratio; ROC, receiver operating characteristic.

The ROC curve was used to analyze the superiority index for determining chemotherapy ineligibility for the elderly. We selected eight indices: age, ECOG PS, number of previous regimens, BMI, NLR, PLR, CAR, and CA19-9; the AUC values were 0.60 (*p* = 0.43), 0.53 (*p* = 0.80), 0.59 (*p* = 0.47), 0.70 (*p* = 0.11), 0.79 (*p* = 0.02), 0.58 (*p* = 0.54), 0.89 (*p* = 0.002), and 0.55 (*p* = 0.70), respectively (Figure 2). Accordingly, we selected CAR and NLR as indices for detecting eligible patients, given that AUC values were high and statistically significant. We also analyzed index cut-off values, i.e., 0.45 and 2.8. In our study, there were seven patients in the ineligible group. In those groups, there were no significant differences in the ECOG PS performance status, the number of metastatic sites, the presence of liver metastasis, the number of previous regimens, and previous irinotecan use. For reference, the analysis of the non-elderly group also showed that PLR, CAR, and CA19-9 were useful based on AUC values (AUC; 0.80, 0.783, and 0.808, respectively).

### 3.5. Distributions of CAR and NLR between Eligible and Ineligible Patients in the Elderly Group

**Figure 3:**
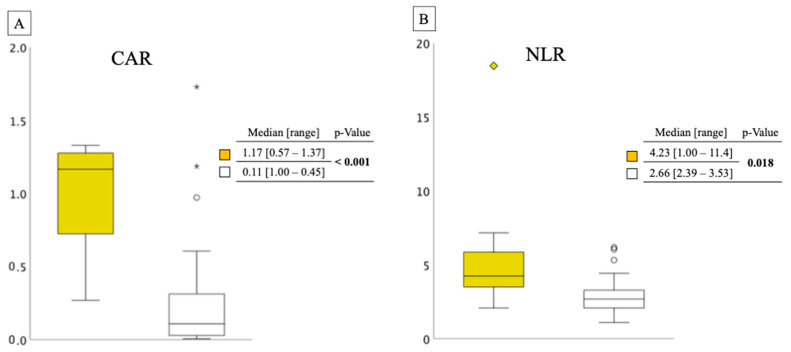
(**A**) This was a box-and-whisker diagram of two groups. The vertical axis showed the width of the CAR and the distribution of the two groups. ◆, ★ and ○ in the figure indicate outliers. CAR distribution between ineligible and eligible cases in the elderly group. Yellow box: ineligible group; white box: eligible group. (**B**) NLR distribution between ineligible and eligible cases in the elderly group. Yellow box: ineligible group; white box: eligible group. CAR, C-reactive protein/albumin ratio; NLR, neutrophil/lymphocyte ratio.

Figure 3 presents the distributions of CAR and NLR between the two groups. Ineligible cases had significantly higher CAR and NLR scores than eligible cases. The median CAR and NLR scores in the ineligible group were 1.17 and 4.23 (*p* < 0.001 and *p* = 0.018, respectively). 

## 4. Discussion

In the present study, we examined two main issues: the safety and efficacy of nal-IRI+5-FU/LV and chemotherapy eligibility indicators in elderly patients with URPC. We retrospectively examined patients with URPC who were treated with nal-IRI+5-FU/LV. Although a high number of patients in the elderly group had poor physical conditions, such as organ function, there were no significant differences in safety and efficacy between the elderly and non-elderly groups. Moreover, focusing on the elderly group only, we investigated potential indicators to determine patients eligible to undergo nal-IRI+5-FU/LV. We found that CAR and NLR could be used as indicators for eligibility. In such cases, eligibility for chemotherapy should be thoroughly reviewed. 

The enrollment of elderly patients in clinical trials is frequently hindered owing to various factors, and the NAPOLI-1 trial included only 14 patients over 75 years of age (12.0%) [2,15]. A lack of elderly patients in clinical trials remains one of the most important problems in treating elderly patients. Herein, the elderly and non-elderly groups tended to have similar OS, PFS, and adverse events. Although the elderly group experienced significantly more comorbid organ damage than the non-elderly group, the similar level of adverse events between the two groups was attributed to dose adjustment from the first initiation dosage. Regarding the characteristics of the non-elderly group, a high number of patients had undergone pretreatment irinotecan, along with several treatment regimens. Using data from a subgroup analysis of a small number of patients in the NAPOLI-1 trial and another retrospective study, patients exhibiting these characteristics were defined as a population with poor response to the nal-IRI [16]. There was a previous report in the United States database which showed that history of irinotecan use, number of prior treatments, and NLR contributed to OS [17]. In the present study, OS was significantly worse in the elderly group when prognostic factors were unified and compared; this indicated that the possibility of underestimation of OS for the non-elderly group could not be ruled out because of the large number of cases in the non-elderly group with backgrounds of poor efficacy of nal-IRI+5-FU/LV reported previously [2]. However, in the case of dosage adjustments, nal-IRI+5-FU/LV is a safe and effective treatment for elderly patients. 

Precisely treating elderly patients without overtreatment or undertreatment can be challenging. As mentioned above, several patients were recommended for BSC based on age alone [6]. Conversely, the overt pursuit of treatment might worsen the general condition, and the prognosis might be shortened owing to more patients with a poor general condition when compared with non-elderly patients. Therefore, we analyzed indicators to evaluate the eligibility of patients for initiating nal-IRI+5-FU/LV therapy. We selected various factors, such as age, PS, BMI, inflammatory nutritional factors, previously reported prognostic indicators, and CA19-9. ROC curve analysis determined that CAR and NLR were notable discriminative indicators of eligibility (AUC; 0.89 and 0.79, respectively). These results suggest that CAR and NLR can be used as objective indicators to identify patients eligible for chemotherapy. Conversely, we also performed a similar analysis in the non-elderly group and found that PLR, CAR, and CA19-9 were useful indicators of eligibility (AUC; 0.80, 0.783, and 0.808). Moreover, a significant difference was observed between the two groups. In the current study, NLR might be specific to the elderly, with CAR found to be more sensitive in the elderly. NLR has been previously reported as a prognostic factor, although its implication among elderly patients was unclear. CAR and NLR are inflammatory nutritional factors that reflect the nutritional status and have been reported as prognostic factors for URPC and other cancers [7,8,18,19]. These two factors reflect inflammatory cytokines, such as interleukin-6, and may function as simple indices highlighting certain aspects of cancer cachexia, as previously reported [20]. Elderly patients with URPC and cancer cachexia were deemed a poor prognosis population and found to experience a high rate of chemotherapy-induced toxicity, such as neutropenia [19]. Therefore, improving the nutritional status of patients with URPC exhibiting cancer cachexia is crucial, and some trials have been conducted to improve this status. In addition, trials have investigated the efficacy of a ghrelin receptor agonist, anamorelin, which was approved in Japan for treating cancer cachexia, along with functional foods such as active hexose-correlated compounds [21,22]. These indicators should be considered when treating elderly patients using nal-IRI+5-FU/LV therapy. A previous study reported a nomogram for evaluating prognostic prediction in the second-line treatment of PC [23]; however, the second-line treatment regimens varied, and nal-IRI+5-FU/LV cases were not evaluated independently. However, NLR was considered a prognostic indicator in the present report. 

The evaluation of eligibility for chemotherapy is one of the most critical challenges in cancer treatment, even when considering cancers other than PC. Prognostication is an important issue when considering eligibility. In general, as in our study, patients with a prognosis of <3 months should be considered ineligible for chemotherapy. Various attempts have been made to predict prognosis [24]. In general, the prognosis is often assumed to be longer, according to the judgment of the attending physician. Recent trials have examined prognosis within three months using artificial intelligence [25,26]. The development of objective indicators can be expected in the future. Geriatric assessment is also an important issue in terms of evaluating indications for cancer treatment in elderly patients. Various factors are widely known as the tools for predicting chemotherapy toxicity and prognosis, such as the Cancer and Aging Research Group Chemotherapy Toxicity Calculator (CARG) and Geriatric 8 (G8). However, the tools have not yet been widely used in practice at the level of adjusting the treatment plan. This is one of the areas in which future evidence accumulation is expected [27].

Our study has some limitations. This was a retrospective study, and only a few patients were included. In addition, there were several differences in the patients’ backgrounds between the elderly and non-elderly groups.

## 5. Conclusions

In conclusion, despite the high number of elderly patients with aggravated kidney and hepatic function, there were no significant differences in OS and PFS between elderly and non-elderly patients treated with the nal-IRI+5-FU/LV. Considering the elderly group, CAR and NLR could be potential indicators to determine eligibility for nal-IRI+5-FU/LV therapy. However, various limiting factors persist, and additional investigations with validated datasets are warranted.

## Figures and Tables

**Table 1:** Patient characteristics.

Characteristics	Elderly *n* = 32 (%)	Non-Elderly *n* = 53 (%)	*p*-Value
Age (years)	78.5 (75–88)	65 (48–74)	−
Median [range]			
Sex	17/15 (53/47)	32/21 (60/40)	0.65
Male/Female			
ECOG PS	9/23 (28/72)	20/33 (38/62)	0.48
0/≥1			
BMI	20.3 (15.9–25.6)	20.5 (13.7–35.1)	−
Median [range]			
ALBI score	−2.38 (−2.90–−1.52)	−2.57 (−3.29–−1.17)	0.02
Median [range]			
CCR	52.5 (35.1–95.6)	78.9 (26.4–175.2)	<0.01
Median [range]			
Location	13/12/7 (41/38/21)	29/16/8 (55/30/15)	0.56
Head/body/tail			
Status	7/25 (22/78)	4/49 (7/93)	0.09
Locally/recurrence or unresectable			
No. of metastatic site	1.5 (0–4)	2 (0–4)	0.01
Median [range]			
Liver metastasis	11/21 (34/66)	35/18 (66/34)	0.01
Yes/No			
Lung metastasis	10/22 (31/69)	19/34 (36/64)	0.81
Yes/No			
Peritoneum metastasis	12/20 (38/62)	23/30 (43/57)	0.65
Yes/No			
Ascites	9/23 (28/72)	22/31 (42/58)	0.25
Yes/No			
No. of previous regimens	23/9 (72/28)	24/29 (45/55)	0.02
1/≥2			
Previous irinotecan regimen	2/30 (6/94)	18/35 (34/66)	<0.01
Yes/No			
Dose reduction at initiation	15/17 (47/53)	6/47 (11/89)	<0.01
Yes/No			

ECOG PS, Eastern Cooperative Oncology Group performance status; BMI, body mass index; ALBI score, Albumin-Bilirubin score; CCR, creatinine clearance; No., number.

**Table 2:** Adverse events in the elderly and non-elderly groups.

	Elderly		Non-Elderly			
	CTCAE Grade				*p*-Value	
	Any Grade (%)	Grade 3–4 (%)	Any Grade (%)	Grade 3–4 (%)	Any Grade (%)	Grade 3–4 (%)
Hematologic						
Neutropenia	11 (34%)	10 (31%)	14 (26%)	11 (21%)	0.47	0.31
Anemia	1 (3%)	1 (3%)	3 (6%)	3 (6%)	1.00	1.00
Platelet decreased	1 (3%)	0	1 (2%)	1 (2%)	1.00	1.00
Non-hematological						
Febrile neutropenia	1 (3%)	1 (3%)	0	0	0.38	0.38
Anorexia	11 (34%)	0	12 (23%)	1 (2%)	0.31	1.00
Nausea	3 (9%)	0	10 (19%)	0	0.35	1.00
Diarrhea	11 (34%)	1 (3%)	12 (23%)	1 (3%)	0.31	1.00
Fatigue	6 (18%)	0	10 (19%)	0	1.00	1.00
Edema	0	0	2 (4%)	0	0.52	1.00
Infusion reaction	0	0	1 (2%)	1 (2%)	1.00	1.00
Infection	0	0	1 (2%)	0	1.00	1.00
AST/ALT Elevation	0	0	3 (6%)	1 (2%)	0.29	1.00
Cholangitis	1 (3%)	0	1 (2%)	1 (2%)	1.00	1.00
Hypokalemia	1 (3%)	1 (3%)	1 (2%)	1 (2%)	1.00	1.00
Hyponatremia	1 (3%)	1 (3%)	2 (6%)	0	1.00	0.38
Mucositis oral	1 (3%)	0	1 (2%)	0	1.00	1.00
Constipation	1 (3%)	1 (3%)	1 (2%)	0	1.00	0.38
Hiccups	1 (3%)	0	0	0	0.38	1.00
Confusion	1 (3%)	1 (3%)	0	0	0.38	0.38
Alopecia	1 (3%)	0	1 (2%)	0	1.00	1.00

CTCAE grade, Common Terminology Criteria for Adverse Events; AST, aspartate aminotransferase; ALT, alanine aminotransferase.

## Data Availability

Not applicable.

## References

[1] Park W., Chawla A., O’Reilly E.M. (2021). Pancreatic cancer: A review. JAMA.

[2] Macarulla T., Blanc J.F., Wang-Gillam A., Chen L.T., Siveke J.T., Mirakhur B., Chen J., de Jong F.A. (2019). Liposomal irinotecan and 5-fluorouracil/leucovorin in older patients with metastatic pancreatic cancer—A subgroup analysis of the pivotal NAPOLI-1 trial. J. Geriatr. Oncol..

[3] Conroy T., Desseigne F., Ychou M., Bouché O., Guimbaud R., Bécouarn Y., Adenis A., Raoul J.L., Gourgou-Bourgade S., de la Fouchardière C. (2011). FOLFIRINOX versus gemcitabine for metastatic pancreatic cancer. N. Engl. J. Med..

[4] Von Hoff D.D., Ervin T., Arena F.P., Chiorean E.G., Infante J., Moore M., Seay T., Tjulandin S.A., Ma W.W., Saleh M.N. (2013). Increased survival in pancreatic cancer with nab-paclitaxel plus gemcitabine. N. Engl. J. Med..

[5] Wang-Gillam A., Li C.P., Bodoky G., Dean A., Shan Y.S., Jameson G., Macarulla T., Lee K.H., Cunningham D., Blanc J.F. (2016). Nanoliposomal irinotecan with fluorouracil and folinic acid in metastatic pancreatic cancer after previous gemcitabine-based therapy (NAPOLI-1): A global, randomised, open-label, phase 3 trial. Lancet.

[6] Kuroda T., Kumagi T., Yokota T., Azemoto N., Hasebe A., Seike H., Nishiyama M., Inada N., Shibata N., Miyata H. (2017). Efficacy of chemotherapy in elderly patients with unresectable pancreatic cancer: A multicenter review of 895 patients. BMC Gastroenterol..

[7] Hang J., Xue P., Yang H., Li S., Chen D., Zhu L., Huang W., Ren S., Zhu Y., Wang L. (2017). Pretreatment C-reactive protein to albumin ratio for predicting overall survival in advanced pancreatic cancer patients. Sci. Rep..

[8] Zhou Y., Wei Q., Fan J., Cheng S., Ding W., Hua Z. (2018). Prognostic role of the neutrophil-to-lymphocyte ratio in pancreatic cancer: A meta-analysis containing 8252 patients. Clin. Chim. Acta.

[9] Ikoma T., Shimokawa M., Matsumoto T., Boku S., Yasuda T., Shibata N., Kurioka Y., Takatani M., Nobuhisa T., Namikawa T. (2023). Inflammatory prognostic factors in advanced or recurrent esophageal squamous cell carcinoma treated with nivolumab. Cancer Immunol. Immunother..

[10] Li W., Tao L., Lu M., Xiu D. (2018). Prognostic role of platelet to lymphocyte ratio in pancreatic cancers: A meta-analysis including 3028 patients. Medicine.

[11] Cockcroft D.W., Gault M.H. (1976). Prediction of creatinine clearance from serum creatinine. Nephron.

[12] Deng M., Ng S.W.Y., Cheung S.T., Chong C.C.N. (2020). Clinical application of albumin-bilirubin (ALBI) score: The current status. Surgeon.

[13] Hiraoka A., Michitaka K., Kumada T., Izumi N., Kadoya M., Kokudo N., Kubo S., Matsuyama Y., Nakashima O., Sakamoto M. (2017). Validation and potential of albumin-bilirubin grade and prognostication in a nationwide survey of 46,681 hepatocellular carcinoma patients in Japan: The need for a more detailed evaluation of hepatic function. Liver Cancer.

[14] Johnson P.J., Berhane S., Kagebayashi C., Satomura S., Teng M., Reeves H.L., O’Beirne J., Fox R., Skowronska A., Palmer D. (2015). Assessment of liver function in patients with hepatocellular carcinoma: A new evidence-based approach-the ALBI grade. J. Clin. Oncol..

[15] Talarico L., Chen G., Pazdur R. (2004). Enrollment of elderly patients in clinical trials for cancer drug registration: A 7-year experience by the US Food and Drug Administration. J. Clin. Oncol..

[16] Kawakami T., Todaka A., Oshima K., Fushiki K., Hamauchi S., Tsushima T., Yokota T., Onozawa Y., Yasui H., Yamazaki K. (2023). Biomarker analysis for patients with pancreatic cancer treated with nanoliposomal irinotecan plus 5-fluorouracil/leucovorin. BMC Cancer.

[17] Barzi A., Miksad R., Surinach A., Corvino F.A., Wang S., Torres A.Z., Mamlouk K., Pulgar S., Valderrama A., Bekaii-Saab T. (2020). Real-World Dosing Patterns and Outcomes of Patients with Metastatic Pancreatic Cancer Treated with a Liposomal Irinotecan Regimen in the United States. Pancreas.

[18] Zhang J., Zhang C., Li Q., Zhang J., Gu X., Zhao W., Chen M., Liu M., Zhang Z., Liao X. (2019). C-reactive protein/albumin ratio is an independent prognostic predictor of survival in advanced cancer patients receiving palliative care. J. Palliat. Med..

[19] Koga F., Kawaguchi Y., Shimokawa M., Murayama K., Nakashita S., Oza N., Ureshino N., Takahashi H., Ueda Y., Nakazawa J. (2022). Gemcitabine plus nab-paclitaxel in older patients with metastatic pancreatic cancer: A post-hoc analysis of the real-world data of a multicenter study (the NAPOLEON study). J. Geriatr. Oncol..

[20] Burgassi F., Paillaud E., Poisson J., Bousquet G., Pamoukdjian F. (2021). Prognostic value of prospective longitudinal CRP to albumin ratio among older outpatients with cancer. Cancers.

[21] Takeda T., Sasaki T., Suzumori C., Mie T., Furukawa T., Yamada Y., Kasuga A., Matsuyama M., Ozaka M., Sasahira N. (2021). The impact of cachexia and sarcopenia in elderly pancreatic cancer patients receiving palliative chemotherapy. Int. J. Clin. Oncol..

[22] Garcia J.M., Boccia R.V., Graham C.D., Yan Y., Duus E.M., Allen S., Friend J. (2015). Anamorelin for patients with cancer cachexia: An integrated analysis of two phase 2, randomised, placebo-controlled, double-blind trials. Lancet Oncol..

[23] Hashimoto D., Satoi S., Yamamoto T., Yamaki S., Ishida M., Ryota H., Sakaguchi T., Hirooka S., Inoue K., Sekimoto M. (2021). Nutritional impact of active hexose-correlated compound for patients with resectable or borderline-resectable pancreatic cancer treated with neoadjuvant therapy. Surg. Today.

[24] Hsu C.C., Liu K.H., Chang P.H., Chen P.T., Hung C.Y., Hsueh S.W., Yeh K.Y., Chen Y.Y., Lu C.H., Hung Y.S. (2020). Development and validation of a prognostic nomogram to predict survival in patients with advanced pancreatic cancer receiving second-line palliative chemotherapy. J. Gastroenterol. Hepatol..

[25] Hui D., Paiva C.E., Del Fabbro E.G., Steer C., Naberhuis J., van de Wetering M., Fernández-Ortega P., Morita T., Suh S.Y., Bruera E. (2019). Prognostication in advanced cancer: Update and directions for future research. Support. Care Cancer.

[26] Zachariah F.J., Rossi L.A., Roberts L.M., Bosserman L.D. (2022). Prospective comparison of medical oncologists and a machine learning model to predict 3-month mortality in patients with metastatic solid tumors. JAMA Netw. Open.

[27] Chapman A.E., Elias R., Plotkin E., Lowenstein L.M., Swartz K. (2021). Models of Care in Geriatric Oncology. J. Clin. Oncol..

